# A Soft Material Flow Sensor for Micro Air Vehicles

**DOI:** 10.1089/soro.2019.0130

**Published:** 2021-04-16

**Authors:** Johan Sundin, Katherine Kokmanian, Matthew K. Fu, Shervin Bagheri, Marcus Hultmark

**Affiliations:** ^1^ Department of Engineering Mechanics, Linné FLOW Centre, Royal Institute of Technology, Stockholm, Sweden.; ^2^ Mechanical and Aerospace Engineering Department, Princeton University, Princeton, New Jersey, USA.; ^3^ Department of Civil and Environmental Engineering, Stanford University, Stanford, California, USA.

**Keywords:** soft sensor, flow measurements, micro air vehicles, stretchable electronics, PDMS

## Abstract

To control and navigate micro air vehicles (MAVs) efficiently, there is a need for small, lightweight, durable, sensitive, fast, and low-power airspeed sensors. When designing sensors to meet these requirements, soft materials are promising alternatives to more traditional materials due to the large deformations they can withstand. In this article, a new concept of a soft material flow sensor is presented based on elastic filament velocimetry, which fulfills all necessary criteria. This technique measures flow velocity by relating it to the strain of a soft ribbon suspended between two static supports and subjected to a flow of interest. The ribbon is manufactured from polydimethylsiloxane and can be made piezoresistive by the addition of silver nanowires. With the described manufacturing method, the sensor can be made using common laboratory tools, outside of a clean room, significantly reducing its complexity. Furthermore, it can be operated using a simple and lightweight circuit, making it a convenient alternative for MAVs. Using a piezoresistive material allows for the flow velocity to be calibrated to the resistance change of the strained ribbon. Although certain challenges remain unsolved, such as polymer creep, the sensor has demonstrated its ability to measure flow velocities down to 4 m/s in air through experiments. A time-dependent analytical model is also provided. The model shows that the current sensor has a bandwidth of 480 Hz. Most importantly, the sensitivity and the bandwidth of the sensor can be varied strictly by modifying the geometry and the material properties of the ribbon.

## Introduction

With the recent improvements to battery technology and inertial measurement units, there has been increasing interest and availability in small unmanned aerial vehicles, particularly in micro air vehicles (MAVs).^1^ These vehicles, which have masses on the order of 100 g, operate close to the ground^2^ and travel at velocities comparable to their surrounding wind speeds (i.e., around 10 m/s). Consequently, wind, and particularly gusts, can significantly impact the optimal trajectory and stability of the vehicle when flying between two points,^3–6^ even when systems such as Global Positioning System (GPS) are used to estimate ground speed. When performing path optimization, measuring the local wind speed requires an anemometry technique that is not only fast enough to resolve changes in wind speed, but it is also sufficiently durable, small, lightweight, sensitive, and energy efficient^1^ to minimally impact vehicle performance. There are few conventional techniques designed with all these considerations in mind. Pitot-static tubes are a robust but slow method for measuring wind speed and can be used on larger MAVs.^7,8^ However, since they measure dynamic pressure, which scales with velocity as $p\propto U_{\infty }^{2}$, detecting small pressure differences created by low velocities requires large transducers. Thermal techniques, such as conventional hot-wire anemometry, have the requisite sensitivity, form factor and bandwidth, but are far too delicate, expensive, and prone to drift^9^ for practical use.^2^ There also exist some indirect approaches to estimate wind speed based on data from other onboard sensors.^5,6^ However, such methods typically come with limitations such as high uncertainty and time lag, restricting their applicability. Recent improvements in the fabrication of functional soft material sensors have the potential to bridge this gap in sensing capabilities.

Soft material sensors are commonly used to interface with deformable surfaces as they can accommodate the large engineering strains experienced by the surface. Unlike sensors consisting of stiff materials, soft piezoresistive sensors can be used on skin to measure strain^10–12^ or pressure.^13,14^ These types of sensors have been made possible through the development of new, highly deformable, electrically conductive materials,^15,16^ the most common variants of which are aggregates consisting of conductive particles (e.g., silver nanowires [AgNWs],^17–22^ silver microparticles,^23^ graphene,^14,24,25^ or carbon nanotubes^11,12,14^) interspersed within a polymeric binding agent, such as polydimethylsiloxane (PDMS) or polyimide. While these composites derive their electrical conductivity and piezoresistivity from the contact and tunneling between the particulates,^23^ the mechanical properties of the composites are determined mainly by the polymer, allowing them to support exceptionally large strains compared with pure metals (ε on the order of 1).^17,18^ Polymers with liquid metal inclusions constitute another common variant of piezoresistive soft sensors.^26^

Similar sensors have been developed for velocity and shear stress measurements. Most of these are biomimetics, often utilizing cantilever beam-like form factors that rely on the bending moment of a calibrated structure when subjected to flow. Strain-sensitive traces placed on or around the structure can then be used to measure the local strain and correlate it with the flow. Perhaps, the most notable example is the artificial lateral line (ALL), inspired by the neuromasts found on fish, which allow them to sense water velocity.^27^ Neuromasts on fish consist of hair cells embedded in soft cupulae that are typically 0.1 mm in length, whereas engineered versions are on the order of 1 mm. Synthetic ALLs have received considerable attention in recent years, often consisting of stiffer materials such as metal or SU-8,^27,28^ although softer variants have begun to emerge as well.^29–33^ Whiskers are another source of inspiration stemming from biology.^24,29^ In addition to velocity, similar cantilever beam-like sensors have been used to measure wall shear stress.^34–36^ While these sensors rely on soft materials to achieve a high degree of sensitivity, this often comes at the expense of temporal bandwidth.

Other sensor designs utilize doubly supported sensing elements, such as the spider silk-inspired acoustic sensors developed by Zhou and Miles,^37^ the optical fiber described by Stadler *et al.*,^38,39^ and the recently developed elastic filament velocimetry (EFV) sensor.^40,41^ The latter relies on drag from the passing flow to elongate a thin, conductive platinum ribbon suspended between two static supports. The resistance change due to the induced axial strain can be measured via a strain gauge effect and related to the flow velocity. The small dimensions of this platinum ribbon, which are on the order of 100 μm in length and 100 nm in thickness, can be easily obtained using standard microelectromechanical system manufacturing techniques and enable high spatial and temporal resolutions.

**FIG. 1.**
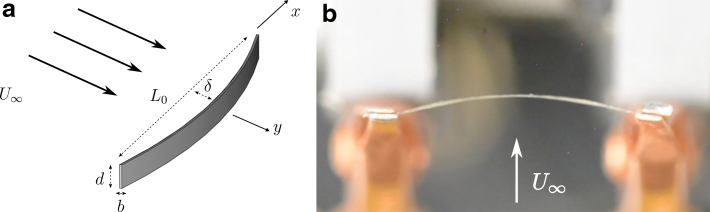
The soft material sensor is shown schematically **(a)** and in a photograph **(b)**. When subjected to fluid flow, the sensor ribbon deflects with a magnitude δ at the centerline. This deformation increases the strain of the ribbon, which in turn changes its resistance since the ribbon is manufactured from a piezoresistive material. The photograph shows the sensor when subjected to airflow of $U_{\infty }=11$ m/s. Color images are available online.

Here, we leverage recent advancements in the fabrication and capability of soft materials to develop a unique flow sensor design with the requisite sensitivity, form factor, and bandwidth for MAV applications. The sensor consists of a soft piezoresistive ribbon that deforms when subjected to passing flow. For a given fluid, the flow velocity can be related to the ribbon's change in resistance. Both a schematic and a photograph of the sensor deflecting in flow are shown in Figure 1, where the unstrained ribbon has a length $L_{0}=15$ mm, width d=300 μm, and thickness b=97 μm. Utilizing soft piezoresistive materials in lieu of conventional materials along with the EFV sensing concept bestows several advantages compared with other strain-based flow sensors.

The key novelty of the sensor lies, to a great extent, in the simplicity of the design, manufacturing, and operation. For cantilever beam-like sensors, additional strain gauges or measurements of deflection are needed, such as piezoresistive elements, piezoelectric elements, or capacitors that deform when the sensor deflects.^27^ Sometimes they rely on complicated structures such as interconnected pillars^30^ or liquid metal plates^33^ enclosed in a soft cupula. These sensors have been made very sensitive, but the fabrication is typically much more intricate than the method described below. For example, the sensors developed by Chen *et al.*^42^ and Kottapalli *et al.*^31,32^ can measure velocities of around 0.1 m/s in air, but their fabrication involves processes that require a clean room. Using the current fabrication process, the sensor can be manufactured using common laboratory tools and low-cost materials without the need for a clean room. It can be operated using a simple and lightweight circuit, making it more accessible to the community compared with most flow sensors. Furthermore, the range of measurable ribbon strains, that is, the sensor rangeability, can be made large since polymers such as PDMS yield at ε∼1, while platinum, which is the metal of choice for many conventional sensors, yields at $\varepsilon ∼10^{-3}$. Because of the wide range of strain it can undergo, the sensor can also withstand significant impulses.

There are also more subtle advantages regarding the theoretical low-range sensitivity of this sensor design. For example, the drag-induced strain in the ribbon scales with velocity as $\varepsilon \propto U_{\infty }^{4∕3}$ and the deflection scales as $\delta \propto U_{\infty }^{2∕3}$. This is in contrast to a cantilever beam of similar size, where the deflection as well as the bending moment scale as $U_{\infty }^{2}$. The smaller exponents of the relationship yield a higher sensitivity for low velocities. The low-range sensitivity can be easily tuned by modifying both the mechanical stiffness of the ribbon (e.g., thickness and elasticity) and the piezoresistivity of the composite.

In the following sections, we describe the derivation of the physical model and the fabrication process of the sensor. Thereafter, we describe an experimental investigation of the sensor behavior. Finally, sensor time scales are evaluated both analytically and with simulations.

## Physical Model

The large aspect ratio of the ribbon greatly simplifies the modeling of the ribbon deflection as well as of the flow around the sensor. A fairly simple model can, therefore, be constructed and used to predict the behavior of the sensor and aid in its design.

With a ribbon of large aspect ratio, the surrounding flow field can be considered quasi-two-dimensional. The behavior of the flow is only dependent on a single parameter, namely the Reynolds number, $Re_{d}=U_{\infty }d∕\nu$, where *d* is the width of the ribbon, $U_{\infty }$ is the flow velocity, and ν is the kinematic viscosity of the fluid. If one considers a flow velocity of 10 m/s, together with a typical air viscosity of $1.5\times 10^{-5}$ m${}^{2}$/s,^43^ the Reynolds number is $Re_{d}=200$. For $Re_{d}$ of this magnitude, inertial effects are dominant and the drag is proportional to $U_{\infty }^{2}$. Drag is commonly described by the drag coefficient,

(1) $$C_{d}=\frac{D}{\frac{1}{2}\rho_{air}dU_{\infty }^{2}},$$

where *D* is the drag force per unit length and $\rho_{air}=1.2$ kg/m^3^ is the air density.^43^ Here, $C_{d}=3.0$ has been prescribed to fit the measured data. Other cross-sectional shapes, such as the cylinder ($C_{d}\approx 1$), hold similar values.^44^ When *Re*_d_∼<1, viscous effects dominate and the drag is then proportional to $U_{\infty }$. This low $Re_{d}$ regimen is where the original EFV sensor operates.^40^

The large aspect ratio of the ribbon enables its deflection to be described by a nonlinear Euler–Bernoulli equation. Considering a stationary ribbon deflection w(x), where *x* is the coordinate in the axial direction, the Euler–Bernoulli equation states that

(2) $$EI\frac{d^{4}w}{dx^{4}}-N\frac{d^{2}w}{dx^{2}}=\frac{1}{2}\rho_{air}dU_{\infty }^{2}C_{d},$$

where *E* is the Young's modulus, $I=db^{3}∕12$ is the area moment of inertia, and *N* is the axial tension. We assume that the axial tension in the ribbon is N=Aσ(ε), where σ is the stress as a function of the engineering strain, $\varepsilon =\Delta L∕L_{0}$ (where ΔL is the length extension), and A=db is the cross-sectional area. The stress σ=Eε can be written in terms of both ε and *E*, where *E* can be a function of ε as well. To account for imperfections in the PDMS, we will allow for a pretension in the ribbon. A positive pretension makes the ribbon appear harder to strain and can be accounted for by adding a term to the axial tension, such that $N=EA\varepsilon +A\sigma_{0}$.

In the region of measurable strains, the displacement is much larger than the thickness. Under these conditions, it is possible to show that the shear force in the material can be neglected.^40^ A model for the sensor behavior can therefore be based solely on the second and third terms in Equation (2).

Since the deflections are much smaller than the length of the sensor, the leading order deflection of the ribbon can be approximated with a parabola, $w=\delta \left(1-4x^{2}∕L_{0}^{2}\right)$, where $x\in \left[-\frac{L_{0}}{2},\frac{L_{0}}{2}\right]$. The parameter δ is the deflection at the center of the ribbon, shown in Figure 1a. This results in

(3) $$\varepsilon =\frac{8\delta^{2}}{3L_{0}^{2}},$$

and Equation (2) becomes

(4) $$\frac{64}{3}\delta^{3}+8\frac{\sigma_{0}}{E}L_{0}^{2}\delta =\frac{L_{0}\rho_{air}dU_{\infty }^{2}C_{d}}{2EA}L_{0}^{3}.$$

Equation (4) is a third-order polynomial in terms of δ, which has a well-defined real solution.^40^ Pretension has a linear contribution, as this part of *N* is independent of δ. The ideal sensor, that is, $\sigma_{0}=0$, results in the scaling $\delta \propto U_{\infty }^{2∕3}$. The deflection also increases with decreasing Young's modulus or thickness.

## Materials and Methods

### Fabrication

**FIG. 2.**
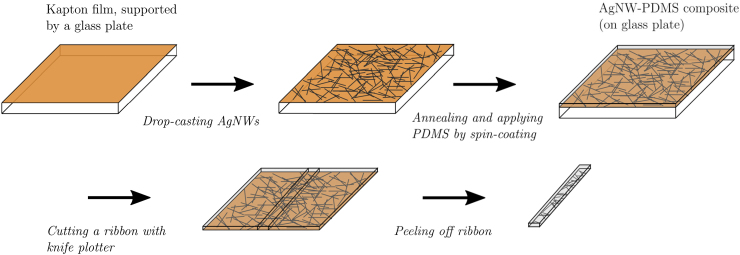
Schematic of the fabrication process of an AgNW-PDMS ribbon. AgNW, silver nanowire; PDMS, polydimethylsiloxane. Color images are available online.

The PDMS ribbon was made conductive through the addition of AgNWs. This procedure, which is schematically outlined in Figure 2, is a common technique used to make polymers conductive.^23^ The properties of a AgNW-PDMS composite are described by Amjadi *et al.*^18^ The AgNWs used here (product number 807923; Sigma–Aldrich) had a diameter of 70 ± 10 nm and a length of 40 ± 5 μm, yielding an aspect ratio of 570. They were in a suspension of ethanol, with a concentration of 5 mg/mL.

**FIG. 3.**
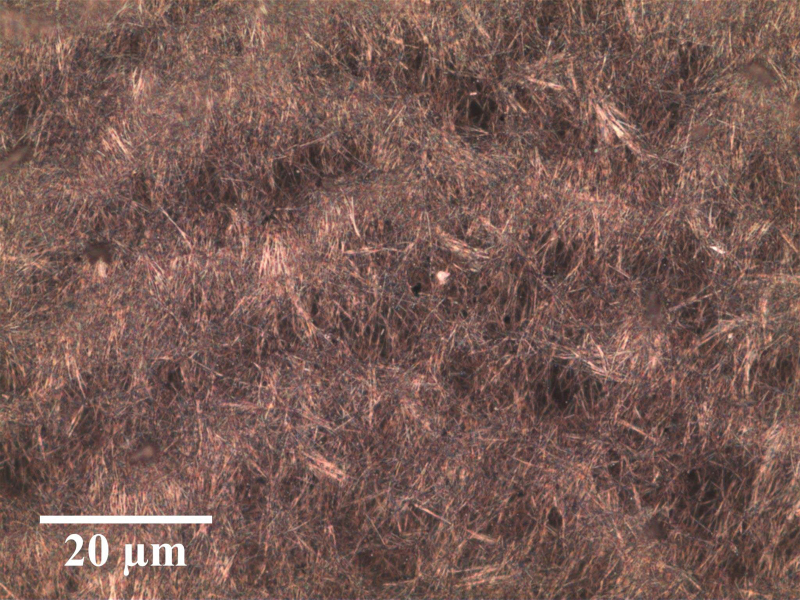
Nanowires photographed with a microscope. Here, the nanowires have been drop-casted onto a glass plate. They have a diameter of ∼70 nm and a length of 40 μm. Color images are available online.

To start the fabrication process, 0.15 mL of the AgNW suspension was drop-casted onto a 20 × 20 mm Kapton film, which had been cleaned with deionized water and isopropyl alcohol. A typical distribution of AgNWs after drop-casting is shown in Figure 3. Although this technique produced slight in-homogeneities in the distribution of AgNWs, this was considered acceptable since their scale is much smaller than the length of the sensor.

The AgNWs were annealed at 200°C for 30 min on a hot plate. PDMS (10:1 elastomer-binding ratio, Sylgard 184) was then spin-coated onto the sample at 1000 rpm, producing a thickness of 97 μm. This resulted in an AgNW-to-PDMS weight ratio of 0.02 (using a specific gravity of PDMS of SG=1.03).^45^ However, the AgNWs were not expected to disperse evenly through the PDMS, but rather to be concentrated on one side of the polymer sheet. The sample was cured at 70°C on a hot plate. This relatively low curing temperature reduces polymer creep, as the polymer cross-links become weaker with an increase in temperature and slip more easily against each other.^46^ Furthermore, this relatively low curing temperature yields a lower Young's modulus for the cured polymer.^47,48^ A ribbon was cut from the resulting film using xurography, that is, with a knife plotter. It has been shown that such a technique can accurately produce widths as small as 200 μm.^49^ Following separation from the film, the ribbon was conditioned by stretching it to slightly above the measuring range several times to reduce hysteresis. More detailed studies of the hysteretic behavior of AgNW-PDMS composites have been performed by Hu *et al.*,^17^ Amjadi *et al.*,^18^ and Zhang *et al.*^23^ The resistance of the ribbon was ∼100Ω; for example, applying a current of 1 mA through the ribbon would require 0.1 mW of electrical power.

### Mechanical and electrical characterizations

To apply the modeled relation between the strain and the velocity of a sensor, Equation (4), the stress–strain and the resistance–strain relations of the ribbon must be characterized. This is equivalent to determining the Young's modulus and the corresponding parameter for the resistance, namely the gauge factor (*GF*). In practice, these properties can also be found by tuning the model until it fits the experimental calibration curve. However, separate experimental tests were performed to measure these values before data collection.

**FIG. 4.**
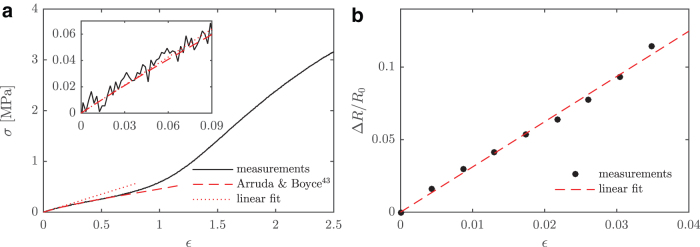
The relation between engineering strain and stress **(a)** and nondimensional resistance **(b)**. Stress relates to the strain by the Young's modulus, *E*, whereas resistance relates to the strain by the gauge factor (*GF*). For small strains, the stress–strain (*inset* in **a**) and the resistance–strain relations are linear, yielding E=0.71 MPa and *GF* = 3.1. Color images are available online.

The stress–strain relation was measured by performing a tensile test (Instron 5865 Universal Testing System with 1 kN range static load cell with 0.5% reading accuracy at 0.1% of full scale). It should be noted that the engineering strain, and not the true strain, was measured. The test was conducted on a sheet of PDMS with a width of 12 mm and the same thickness as the sensor ribbon at an elongation rate of 1 in/min. The results of this test are plotted in Figure 4a and can be seen to consist of two regions. In the first region, up to ε≈1, the composite material resembles a perfect elastomer. At higher strains, the polymers slide against each other, absorbing mechanical work and stiffening the material.^47^ This nonlinear behavior is similar to spider silk—a property that increases the robustness of spider webs.^50^ Due to the low volume ratio of AgNWs in the AgNW-PDMS composite, it was assumed that the composite has the same Young's modulus as pure PDMS. However, the presence of AgNWs in the surface of the sheet may increase the viscoelasticity since viscoelasticity generally increases with particle concentration.^51^

The stress–strain relation in the first region was assumed to be similar to the analytical model developed by Arruda and Boyce^52^ and used for PDMS by Qin *et al.*^53^ This model is shown, together with the measurements, in Figure 4a. For the strains of interest (0<ε<0.03), the model is well approximated by a linear function corresponding to a Young's modulus E=0.71 MPa. This is illustrated in the inset of Figure 4a. This value will therefore be used for the remainder of the analysis.

It has been reported that applying PDMS by spin coating, as was done here, can affect the stress–strain relation, especially for thicknesses smaller than 200 μm.^54^ A thinner sample requires a higher rotational speed, which applies a larger shear stress on the polymers. This aligns the polymers, increasing the Young's modulus of the spin-coated sheet.

Strain is related to the electrical resistance through the strain gauge equation,

(5) $$\frac{\Delta R}{R_{0}}=GF\varepsilon ,$$

where $\Delta R∕R_{0}$ is the relative resistance change and *R*_0_ is the resistance of the unstrained ribbon. For small strains, the relation is linear and *GF* is constant. However, *GF* is seen to increase with strain for larger strain values.^17^ It should be noted that this study focuses on the linear regimen, where the recorded relation is shown in Figure 4b. Results of Hu *et al.*^17^ also indicate that *GF* decreases with increasing AgNW concentration, which therefore should be kept low. However, there exists a threshold below which the sensor response does not remain stable. It was found that the current concentration was necessary to obtain a stable signal.

## Results

The model developed in the previous section, Equation (4), was validated by exposing the sensor to flow velocities in the range of 0 to 11 m/s. This was done by positioning the sensor at the centerline of a low turbulence rectangular air jet and measuring the resistance of the sensor.

**FIG. 5.**
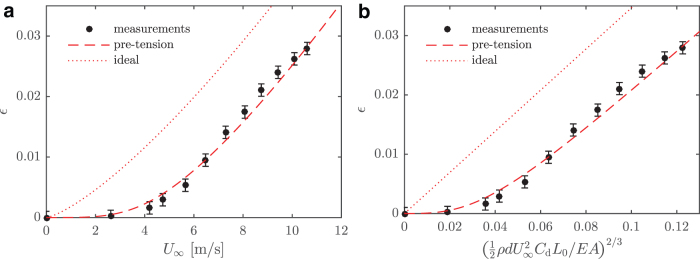
Measured strain–velocity relation with dimensional **(a)** and nondimensional velocity **(b)**. Both measurements and models are shown. For the model, pretension can be included to account for imperfections in the PDMS. Error bars show the root-mean-square deviation of the resistance–strain relation observed in Figure 4b. Color images are available online.

Three consecutive experiments were performed and averaged to calculate the strain of the ribbon. Due to polymer creep, the resistance increased slightly for each data set (∼0.5%), indicating that the current fabrication process can be improved. Flow velocity was measured using a Pitot-static tube (Honeywell HSC TruStability differential pressure transducer, 250 Pa range with 0.25% accuracy). The estimated strain–velocity relation is plotted in Figure 5 along with the modeled strain, Equation (4). Both the ideal model (no pretension) and a model including a pretension of $\sigma_{0}∕E=0.023$ are displayed. If pretension is included, the model has a good agreement with the measurements.

The error bars shown in Figure 5 correspond to the root-mean-square deviation of the linear resistance–strain estimate displayed in Figure 4b. With a more controlled manufacturing method, it is believed that the errors can be reduced further. There are also uncertainties in parameters such as drag coefficient, Young's modulus, and *GF*. These can, in practice, be eliminated by calibration (disregarding nonlinearities).

**FIG. 6.**
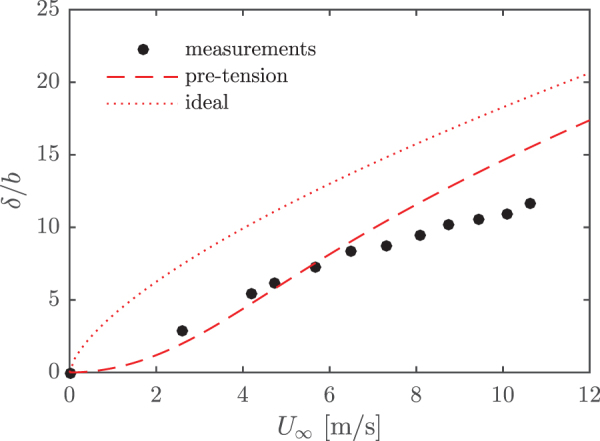
Estimated deflection–velocity relation. The analytical model is also shown, both with and without pretension. Ribbon deflections were determined from photographs. Color images are available online.

From photographs of the sensor, similar to Figure 1b, the ribbon deflection was measured to provide an additional validation of the model. This data set is shown in Figure 6. The correspondence between the model and the measurements is worse here, particularly for larger velocities. These measurements were made with the same sensor as the measurements shown in Figure 5, but at a later time. It is therefore possible that the mechanical properties of the ribbon had changed slightly between the two experiments.

### Dynamics

The stationary behavior of the sensor has been discussed in previous sections. However, characterizing the time scales governing the sensor is of great importance to determine the frequencies it can capture. The time scales of the sensor were determined through analytical estimation and simulations, outlined below. To simplify the expressions, a linearization technique was used.

With both the deflection and the velocity being functions of time, w=w(x,t) and U=U(t), the Euler–Bernoulli equation, Equation (2), can be extended to include the inertia and velocity of the ribbon,^40,41^

(6) $$\begin{array}{cc} & \rho A\frac{\partial^{2}w}{\partial t^{2}}-N\frac{\partial^{2}w}{\partial x^{2}}=\frac{1}{2}\rho_{air}dC_{d}{\left(U-\frac{\partial w}{\partial t}\right)}^{2}\approx \\ & \frac{1}{2}\rho_{air}dC_{d}\left(U^{2}-2U\frac{\partial w}{\partial t}\right), \end{array}$$

where we have assumed that $U\gg \frac{\partial w}{\partial t}$, making the system linear in *w*. Added mass effects have been excluded due to the density of PDMS being much larger than that of air. In fluids of higher density (e.g., water), added mass effects might have to be included in the model.^35^ Similarly, the Basset force term has also been neglected. Maintaining the assumption that the deflected ribbon's shape is a parabola, an additional assumption of quasi-static motion can be made, that is, $w\left(x,t\right)=\phi \left(x\right)\tilde{w}\left(t\right)$, where ϕ(x) represents the spatial shape and $\tilde{w}\left(t\right)$ represents the time-dependent amplitude. For a constant *U*, we impose that $\tilde{w}\left(t\to \infty \right)=\delta$, which is the static deflection, so that $\phi \left(x\right)=1-4x^{2}∕L_{0}^{2}$. Inserting this into Equation (6) and integrating over *x* yields an equation only dependent on time. The system is equivalent to a spring-damper system with a nonlinear spring, similar to the static case, Equation (4), but including the time-dependent terms. This type of second-order differential equation is typically called the Duffing equation.^55^

Due to the nonlinear spring, the natural frequency depends on the amplitude. However, to get a tractable model for the time scales, we can linearize around δ and its corresponding time-independent velocity, $U_{\infty }$, that is, $\tilde{w}=\delta +\tilde{w}\prime$ and $U=U_{\infty }+U\prime$. It is also assumed that $\delta \gg \tilde{w}\prime$ and $U_{\infty }\gg U\prime$. Replacing δ with $\tilde{w}$ in the equation for the strain, Equation (3), and utilizing Equation (4) to simplify Equation (6) results in

(7) $$m\frac{d^{2}\tilde{w}\prime }{dt^{2}}+c\frac{d\tilde{w}\prime }{dt}+k\tilde{w}\prime =F,$$

where $m=\frac{2}{3}L_{0}\rho A$, $c=\frac{2}{3}L_{0}\rho_{air}dU_{\infty }C_{d}$, $k=8\frac{\sigma_{0}A}{L_{0}}+64\frac{EA}{L_{0}^{3}}\delta^{2}$, and $F=L_{0}\rho_{air}dC_{d}U_{\infty }U\prime$. Since this equation describes a linear spring-damper system, the natural frequency and the damping ratio can be computed using the following expressions, respectively^56^:

(8) $$\omega_{n}=\sqrt{\frac{k}{m}}and\zeta =\frac{c}{2\sqrt{km}}.$$

With $U_{\infty }=10$ m/s, δ calculated using Equation (4) and the geometric length scales previously given, it was found that $\omega_{n}=1970$ rad/s and ζ=0.091. Since ζ<1, the sensor is underdamped. Viscoelasticity might increase the damping, but this is beyond the scope of the current article.

**FIG. 7.**
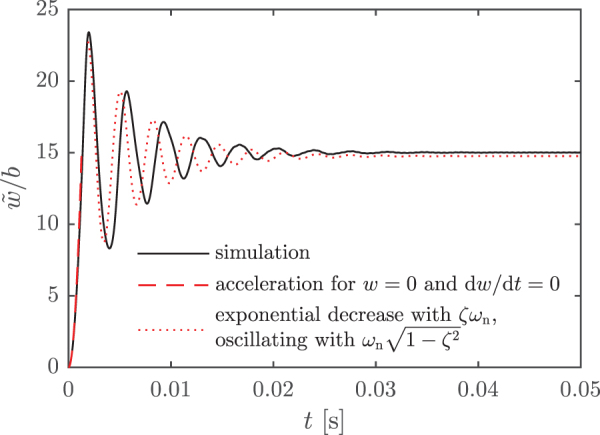
Results of a FEM simulation of the sensor for a step input of $U_{\infty }=10$ m/s, together with analytical predictions. At short times, forces due to displacement and velocity can be neglected, and the acceleration is similar to that at t=0 (*dashed line*). At larger times, the damping sets the time scale for the response, $1∕\left(\zeta \omega_{n}\right)$, and the displacement oscillates with an angular frequency close to $\omega_{n}$ (*dotted line*). Color images are available online.

Assumptions of the model were validated by comparing them with the results of a simulation. The simulation was performed using a finite element method (FEM) algorithm with truss elements^57^ for a step input, going from $U_{\infty }=0$ to 10 m/s, and using the ribbon dimensions above. Large deflections were accounted for by taking geometric nonlinearities into consideration. Furthermore, a rotationally invariant lumped mass matrix was used for the inertia. The ribbon deflections from both the simulation and the analytical results are shown in Figure 7, and the results are in good agreement. At short times, forces due to displacement and velocity are negligible, so that the acceleration of the ribbon is determined by its mass and the initial forcing [first and second to last terms of Eq. (6)]. At larger times, damping becomes important, and the deflection approaches the steady state exponentially with a time scale $1∕\left(\zeta \omega_{n}\right)$, while it oscillates with an angular frequency $\omega_{n}\sqrt{1-\zeta^{2}}$. This is shown by the solution to Equation (7) for U′=0.^56^

By taking the Fourier transformation of Equation (7), it is possible to find the transfer function between the forcing, *F*, and $\tilde{w}\prime$. For this second-order system, the amplitude of the transfer function normalized by 1∕k is^56^:

(9) $$\frac{\left|H\left(\omega \right)\right|}{1∕k}=\frac{1}{\sqrt{{\left(1-{\left(\frac{\omega }{\omega_{n}}\right)}^{2}\right)}^{2}+4\zeta^{2}{\left(\frac{\omega }{\omega_{n}}\right)}^{2}}}.$$

**FIG. 8.**
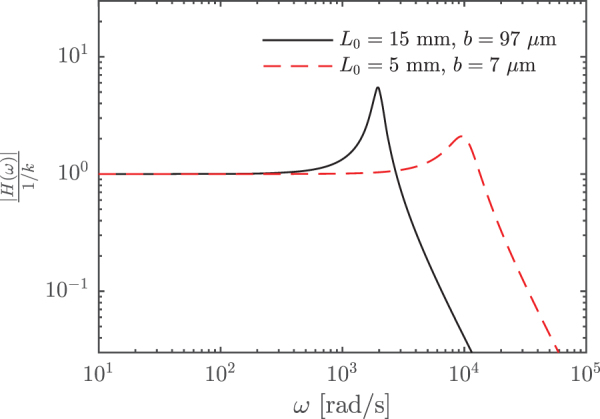
The amplitude of the transfer functions for a velocity $U_{\infty }=10$ m/s and two different ribbon geometries. The width of the ribbon is d=300 μm for both cases. The dimensions used to compute the transfer function shown by the *solid line* represent the current ribbon geometry. Color images are available online.

The Bode magnitude plot for the above transfer function is shown in Figure 8 for two different ribbon geometries. The bandwidth of the system is defined as the frequency where the transfer function has decreased by a factor of $1∕\sqrt{2}$ compared to its static value (at ω=0), which corresponds to −3 dB on a decibel scale. This is found to be $f_{b}=\omega_{b}∕\left(2\pi \right)=480$ Hz. The flat portion of the transfer function terminates at a lower frequency of ∼170 Hz. The low value of ζ is responsible for the relatively significant resonance peak.

For applications related to MAVs, the bandwidth should be compared with frequencies found in the atmospheric boundary layer. Most turbulent fluctuations in the atmospheric boundary layer have frequencies below 10 Hz, but the spectral levels decrease in a continuous manner.^58^ However, if a higher bandwidth and a lower resonance peak are needed, the model shows that the geometry of the ribbon can be modified accordingly. For example, if instead $L_{0}=5$ mm and b=7 μm, then $\omega_{n}=10,000$ rad/s and ζ=0.25 for a velocity of $U_{\infty }=10$ m/s. This yields a bandwidth of $f_{b}=2400$ Hz. The corresponding transfer function is also shown in Figure 8 and is flat up to about 970 Hz. A sensor of these dimensions and properties would also be 23 times more sensitive compared with the one manufactured for this study (assuming a constant *GF*).

The effects of vortex shedding have not been considered here. This phenomenon can be expected when $Re_{d}$ is around 100 and might lead to oscillations in the drag coefficient.^39,44^ We have considered here the average drag coefficient, and further investigations are welcome. However, with an expected Strouhal number close to that of a cylinder, $St=f_{s}d∕U_{\infty }\approx 0.2$, the affected frequencies, $f_{s}$, are above 2000 Hz, which is outside the measuring range of the sensor manufactured.

## Conclusions

A new concept of a soft material flow sensor was developed based on elastic filament velocimetry (EFV). This sensor has been experimentally shown to measure flow velocities down to 4 m/s in air. The sensitivity of the new sensor is only limited by the dimensions of the ribbon and can therefore be adapted to the requirements of specific applications. Both the range of velocities and the time scales will change with geometric modification of the sensor according to the framework presented in this work. In contrast to the original EFV design,^40^ the use of a soft material yields a high durability and enables sensor fabrication without the use of a clean room. This sensor can be made small, lightweight, and requires low power, all of which are crucial characteristics for onboard sensing.

Further investigation regarding polymer creep is warranted since this undesirable phenomenon reduces the reliability of the sensor. This might be solved by improving the PDMS fabrication process or using another polymer. Another solution is to use models which account for polymer creep.^59^ Overall, with the benefits of increased durability, high sensitivity, and simplicity of manufacturing, this novel sensor is a viable alternative to conventional flow sensors. In particular, it is very well suited for providing an onboard estimate of wind speeds for MAVs.
